# Seasonal variation in circulating group 2 innate lymphoid cells in mugwort-allergic asthmatics during and outside pollen season

**DOI:** 10.1186/s13223-018-0229-x

**Published:** 2018-02-09

**Authors:** Qing Miao, Yan Wang, Yong-ge Liu, Yi-xin Ren, Hui Guan, Zhen Li, Wei Xu, Li Xiang

**Affiliations:** Department of Allergy, Beijing Children’s Hospital, Capital Medical School, No. 56 Nanlishi Road, Xicheng District, Beijing, 100045 China

**Keywords:** Asthma, Group 2 innate lymphoid cells, Mugwort, Pollen, House dust mites

## Abstract

**Background:**

Group 2 innate lymphoid cells (ILC2s) are a newly identified cell population with the potent capability to produce Th2-type cytokines in a non-antigen specific manner. Previous study demonstrated that enhanced circulating ILC2s in cat-allergic patient after experimental allergen challenge, whereas the effects of natural allergen exposure on peripheral ILC2s are still unclear. We therefore examined the variations in circulating ILC2s among asthmatic patients sensitized to different allergens in- and outside- pollen season.

**Methods:**

10 patients sensitized to mugwort, 10 patients sensitized to house dust mites (HDM) and 12 healthy controls were recruited into this study. Blood samples were collected from the patients in- and outside- pollens season, 2–3 months apart. ILC2s (Lin-CD127+ CRTH2+) were enumerated by flow cytometry, as well as intracellular IL-5 and IL-13 expression. The levels of IL-5 and IL-13 in supernatants of Lineage- and Lineage+ cells stimulated with IL-25 and/or IL-33 in the presence of IL-2 were measured using a Milliplex human cytokine array kit.

**Results:**

An obvious seasonal increases in percentages of total and IL-13+ ILC2s were observed in patients with mugwort sensitization during natural pollen exposure, however, the percentages of peripheral ILC2s in HDM-allergic patients were not affected significantly. A positive correlation between FeNO and IL-13^+^ILC2s was found in patients sensitized to mugwort. A mixture of IL-33 and IL-25 induced a significant production of IL-13 and IL-5 from Lineage^−^ cells of both mugwort-allergic and HDM-allergic asthmatics. Stimulation with IL-33 alone induced a significantly greater quantity of IL-13 by Lineage-cells from mugwort-allergic asthmatic compared with that from HDM-allergic asthmatics, whereas IL-25 induced a significantly greater amount of IL-5 by the Lineage-cells from mugwort-allergic asthmatic compared with that from HDM-allergic asthmatics.

**Conclusion:**

Within pollen season the frequencies and function profiles of circulating ILC2s among asthmatic children are altered dynamically, which may be closely related to the sensitized type of allergens.

## Background

Asthma is a heterogeneous inflammatory disorders characterized by reversible airway obstruction and bronchial hyper-reactivity and airway inflammation [1, 2]. A nationwide survey in China reported that the prevalence rate of asthma in urban children is 3.02% [3], however, the overall asthma control level is still unsatisfactory [4]. Group 2 innate lymphoid cells (ILC2s) are an important early source of type 2 cytokines and are activated by epithelium-derived alarmins, including interleukin-25 (IL-25), IL-33 and thymic stromal lymphopoietin (TSLP), highlighting the potential critical role of ILC2s in the development of type 2 inflammation [5, 6]. Previously, Doherty et al. have demonstrated that a significant elevation of circulating ILC2s in cat-allergic patients after experimental nasal challenge, suggesting that the effects of allergen exposure on peripheral ILC2s [7]. However, there is little information about the variations in ILC2s during natural allergen exposure. In order to determine whether natural seasonal allergen exposure causes changes in percentage and function of ILC2s in asthma patients, we therefore chose to carry out multiply sampling to observe the changes in peripheral ILC2s at the beginning and outside the pollen season. Unlike the experimental allergen challenge model where a large dose of allergen antigen is given at a single time-point, the strength of seasonal studies design was to observe the whole inflammation process starting from cellular activation and subsequent cytokine production after a continuous allergens exposure within a limited time period. In addition, to further verify whether the role of ILC2s may vary among asthmatics sensitized to different allergens, we investigated the phenotypic and functional characteristics of ILC2s between mugwort and HDM-allergic asthmatics.

## Methods

### Study group

10 subjects with mugwort-allergic asthma and 10 subjects with HDM-allergic asthma were recruited for this study. The diagnosis and selection criteria were based on the global initiative for asthma (GINA). Allergy was confirmed by a positive skin prick testing (SPT) and by the presence of specific IgE. Patients with symptoms of perennial allergy or with concomitant allergy to other seasonal allergens with overlapping time of symptoms occurrence were excluded from this study. If a patient had been taking anti-histamines, steroids, or leukotriene receptor antagonists within 4 weeks, or undergoing immunotherapy for any allergen within the past 3 years prior to the study were excluded. Another 12 healthy, non-atopic children matched for age and sex and with no history of allergy, asthma and other inflammation diseases were enrolled as healthy controls. The research protocol was reviewed and approved by the Beijing Children’s Hospital Human Research Ethic Committee, and informed written consent was obtained from patients’ representatives before enrollment. The study protocol conforms to the ethical guidelines of the Declaration of Helsinki.

### Study design

Blood samples were collected from all enrolled subjects on two occasions through whole season pollen. Test period 1 (August to September, 2016): during pollen season immediately following the appearance of symptoms. At the onset of symptoms, after blood samples collection, all patients were started on treatment with antihistamines. Test period 2 (November, 2016): outside pollen season, when the patients’ symptoms subsided and anti-inflammation treatment was discontinued. At each visit, the patients’ clinical data, including blood eosinophil count, FeNO, and pulmonary function test were record.

### Isolation and preparation of peripheral blood mononuclear cells (PBMCs)

Blood samples were processed within 6 h of sample collection. PBMCs were separated by using Ficoll density gradient solution (HAOYANG, Tianjin, China) at 2000 rpm for 20 min at 4 °C. The PBMCs were collected and washed twice with cold PBS containing 2% fetal calf serum (Gibco, USA) and used for flow cytometric staining (FACS staining).

### ILC2s identification and intracellular staining

**Fig. 1:**
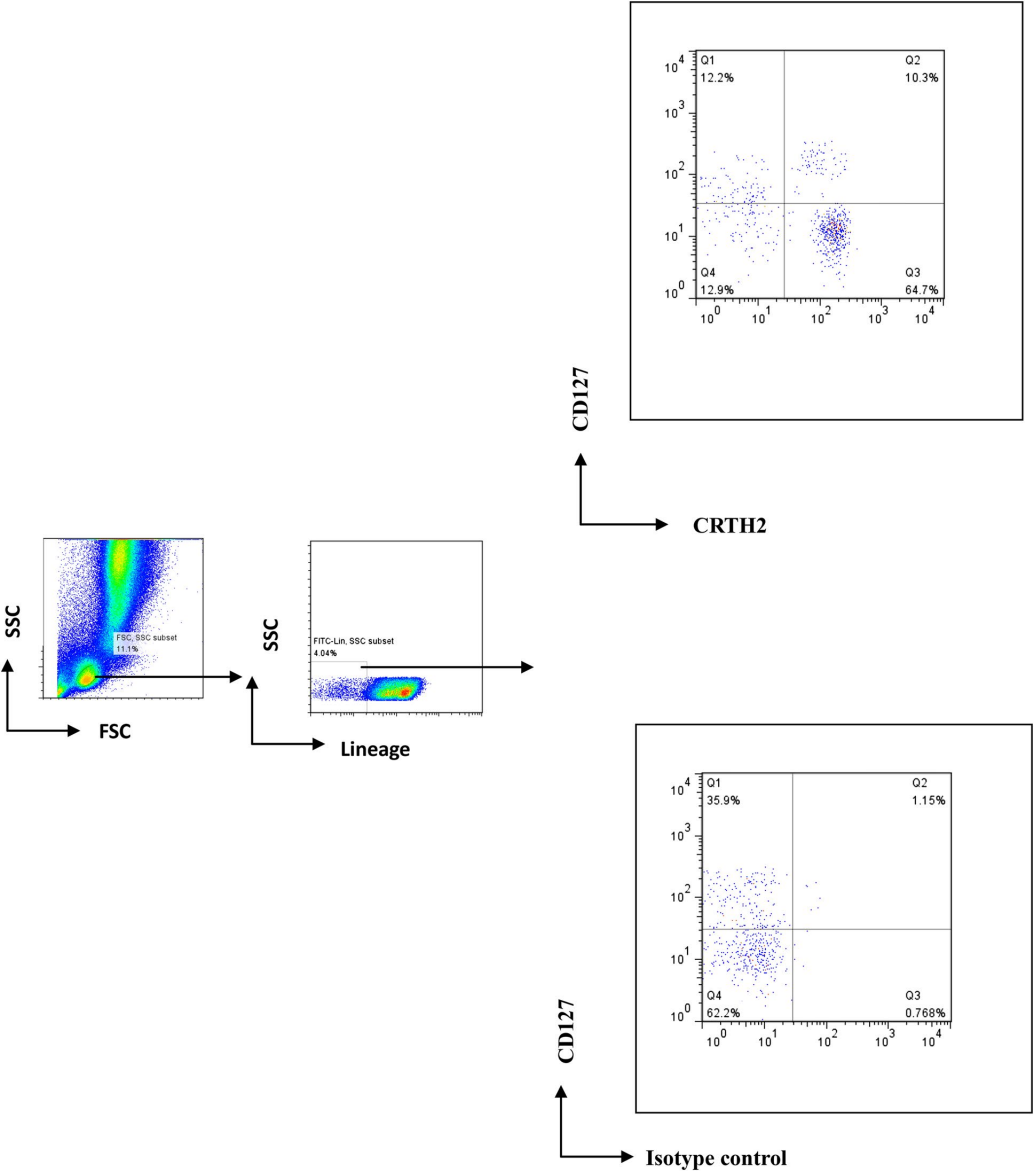
Identification of ILC2s from human peripheral blood using multi FACS staining. Lymphocytes were identified from whole PBMCs (left) and Lineage-negative cells gated (middle). Lineage-negative cells were further assessed for expression of CD127 and CRTH2 (CD294) or isotype control staining (right). ILC2s were identified as Lineage-negative CD127^+^CRTH2^+^ lymphoid like cells. Example shown is an asthmatic patient with pollen sensitization. *FSC* forward scatter. *SSC* side scatter

**Fig. 2:**
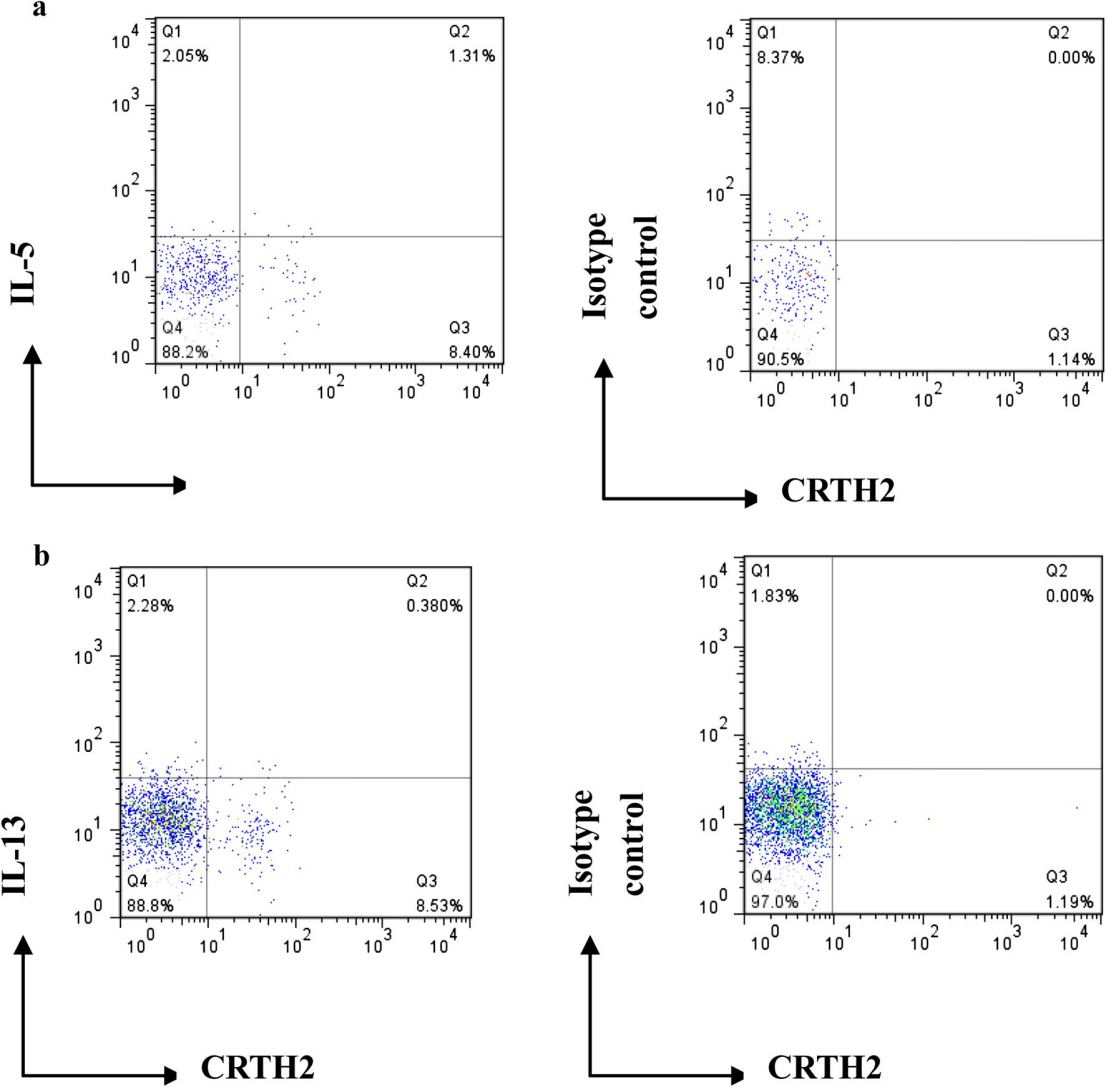
Representative results of intracellular staining of **a** IL-5 and **b** IL-13 expression in ILC2s within mugwort pollen season

Peripheral ILC2s (Lin^−^CD127^+^ CRTH2^+^ cells) in present study were identified as previously reported [8], which were detected by flow cytometric analysis. The PBMCs cell pellet was collected, washed, and then stained with a FITC-conjugated monoclonal antibodies (mAbs) against human Lineage cocktail (CD3, CD14, CD16, CD19, CD34, CD123, CD11c, TCRαβ, and TCRγδ expressed on T cells, monocytes, macrophages, B cells, mast cells, dendritic cells, and hematopoietic progenitor cells), PE-conjugated antibodies against human CD127 (eBioscience, CA, USA), and APC-conjugated antibodies against human CRTH2 (CD294) (Biolegend, CA, USA) at room temperature in the dark for 30 min. We gated on cells lacking Lineage markers and examined expression of CD127 and CRTH2 within lympho-mononuclear region (low side scatter/low forward scatter), and the number of ILC2s is expressed as a percentage of all Lineage negative cells (Fig. 1). For the intracellular staining of cytokines, cells were incubated in Perm/Fix buffer (eBioscience, CA, USA) and optimal concentration of intracellular stains of anti-IL-5-PerCP (BD bioscience) and anti-IL-13-PerCP antibodies (BD bioscience) were added. Gating in the lympho-mononuclear region (low side scatter/low forward scatter) and following acquisition of 100,000 events, data were analyzed using FlowJo program to enumerate intracellular IL-5 and IL-13 levels in ILC2s (Lin-CD45+ CD127+ CRTH2+) (Fig. 2).

### Correlation analysis between ILC2s and asthma clinical parameters

The percentages of IL-13^+^ILC2s were counted among asthma patients subgroups, and its correlations with clinical parameters were analyzed between patients subgroup.

### FACS sorting of ILC2s and in vitro cell culture

Blood derived mononuclear cells were stained with the FITC-Lineage cocktail as described above and then separated into Lineage^+^ and Lineage^−^ cells by using a fluorescence-activated cell sorter (BD FACSAriaII; BD Biosciences). The sorted Lineage^−^ cells were at 5 × 10^4^ cells/mL in 96-well tissue culture plates for 7 days (37 °C, 5% CO_2_) in the presence of medium alone, or IL-2 (20 U/mL) alone, or IL-25 alone (25 ng/mL) (rhIL-25, R&D Systems Inc., Minneapolis, MN, USA, Cat. NO. 8134) plus IL-2 (20 U/mL), or IL-33 alone (25 ng/mL) (rhIL-33, R&D Systems Inc., Minneapolis, MN, USA, Cat. NO. 3625) plus IL-2 (20 U/mL), or their combinations.

### Multiplex analysis of cytokines production in ILC2s cell culture

The concentrations of IL-5 and IL-13 in cell culture supernatants were measured using a Milliplex human cytokine array kit (Millipore, St. Charles, MO, USA) as recommended by the manufacturer.

### Statistical analysis

Statistical analysis was performed using SPSS 19.0 software (SPSS, Chicago, IL, USA) and graphs were generated using the prism software (GraphPad, LaJolla, CA, USA). All data were representative of at least three independent experiments. Results were expressed as the Mean ± SD. Comparison of ILC2% among different groups was performed using unpaired *t* test, and the association between ILC2% and clinical parameters was analyzed using Pearson’s correlation test. All tests were 2-tailed, and *P* value of less than 0.05 was considered as significant.

## Results

### Study subjects

A total of 20 asthmatic children (including 10 patients with mugwort pollen-allergic asthma and 10 patients with HDM-allergic asthma) and 12 healthy controls were enrolled into this study. Detailed information and clinical parameters on these enrolled subjects were shown in Table 1. No significant differences were found in sex, mean age and BMI between patient groups and healthy subjects. However, the differences in FeNO, blood eosinophil counts and FEV1% predicted were statistically significant between two patients subgroups (all *P* < 0.05, respectively).

**Table 1:** The clinical characteristics of enrolled subjects

	Pollen-allergic asthmatics	HDM-allergic asthmatics	Healthy controls	P value
Numbers of patients	10	10	12	–
Boys (%)	5 (50.0)	5 (50.0)	7 (58.3)	–
Age (years)	7.8 ± 3.4	10.2 ± 3.7	9.8 ± 4.0	> 0.05
BMI	15.3 ± 1.6	16.7 ± 1.9	16.0 ± 1.3	> 0.05
FEV1% predicted	77.1 ± 14.6	75.6 ± 13.4	ND	< 0.05
Blood EOS (10^9^/L)	0.51 ± 0.28	0.53 ± 0.23	ND	< 0.05
FeNO (ppb)	56.1 ± 28.1	57.1 ± 24.5	ND	< 0.05

Data expressed as Mean ± SD

*ND* not determined

### Peripheral ILC2s were significantly increased in mugwort pollen-allergic asthmatics compared to HMD-asthmatic patients during pollen season

**Fig. 3:**
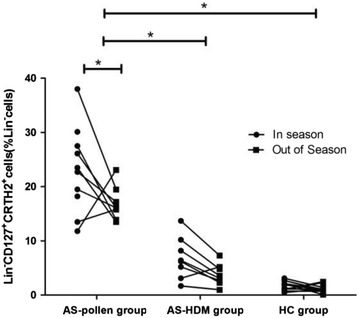
Seasonal changes in the percentage of circulating ILC2s during and outside the pollen season. *P < 0.05. *AS-pollen group* mugwort pollen-allergic asthma group. *AS-HDM group* HDM-allergic asthma group. *HC group* healthy controls

In present study, we first compared the levels of ILC2s in peripheral blood among patients group and healthy controls group during pollen season, showing that the mean number of ILC2s in peripheral blood was significantly increased in asthmatic patients compared to healthy controls (15.01 ± 6.21% vs. 1.69 ± 0.87%, *P* < 0.01). Furthermore, a subgroup analysis indicated the level of ILC2s was higher in subjects with mugwort pollen-allergic asthma compared with those with HDM-allergic asthma (23.09 ± 7.86% vs. 6.84 ± 3.85%, *P* < 0.01). When outside pollen season, it was observed that the number of ILC2s was dramatically higher in pollen-allergic asthma group (11.3 ± 2.45%) compared to the number from health controls (1.32 ± 0.91%), moreover, the difference between mugwort pollen- and HDM-allergic asthmatics was significant (16.9 ± 3.12% vs. 3.76 ± 1.96%, *P* < 0.05) (Fig. 3).

### A seasonal change in percentages of IL-13^+^ILC2, but not IL-5^+^ILC2s, was observed in pollen-allergic asthmatics

**Fig. 4:**
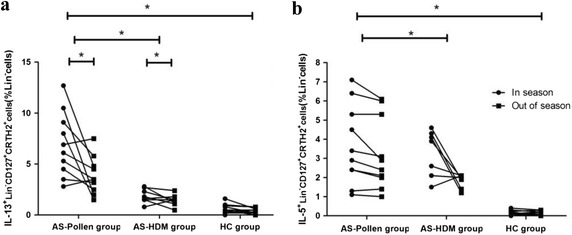
Seasonal changes in **a** IL-13^+^ILC2s **b** IL-5^+^ILC2s during and outside the pollen season. *P < 0.05. *AS-pollen group*: mugwort pollen-allergic asthma group. *AS-HDM group* HDM-allergic asthma group. *HC group* healthy controls

Previous studies demonstrated that ILC2s contribute to production of key cytokines IL-5 and IL-13 in response to epithelium-derived cytokines, such as IL-25 and IL-33 [9, 10]. Accordingly, we further investigated the intracellular cytokine expression of IL-5 and IL-13 in ILC2s. During pollen season, the number of IL-13^+^ILC2s was significantly higher in peripheral blood of pollen-allergic patients (6.94 ± 3.16%) compared to HDM-allergic patients (1.89 ± 0.70%) as well as compared to HCs (0.51 ± 0.50%). When outside pollen season, an obvious decline tendency of IL-13^+^ILC2s was found in patients with pollen-allergic asthma (6.94 ± 3.16% vs. 4.17 ± 1.98%, *P* < 0.05) and in those with HDM-allergic asthma (1.89 ± 0.70% vs. 1.44 ± 0.55%, *P* < 0.05), respectively. However, the percentage of IL-13^+^ILC2s in healthy controls was not affected (0.51 ± 0.50% vs. 0.45 ± 0.30%, *P* > 0.05) (Fig. 4a). A similar analysis was performed to evaluate percentage of the IL-5^+^ILC2s percentage in each group, however, no significant changes were observed in samples from any of the study groups (Fig. 4b). Therefore, IL-13^+^ILC2s were used for the further correlation analysis.

### Circulating IL-13^+^ILC2s numbers were positively correlated with clinical parameters

**Fig. 5:**
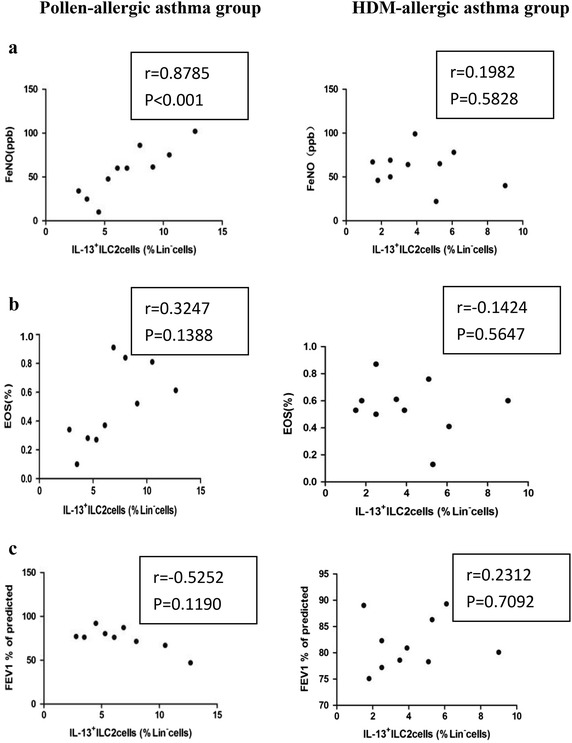
Correlation analysis between IL-13^+^ILC2% levels and clinical parameters among pollen- and HDM-allergic asthmatics. **a** FeNO. **b** Percentage of eosinophils. **c** FEV1% of predicted

A further correlation analysis between IL-13^+^ILC2s levels and clinical parameters, including eosinophils counts, FeNO, FEV1% of predicted was performed. Within the pollen season, a positive correlation between the percentages of circulating IL-13^+^ILC2s and FeNO levels was observed in patients with pollen-allergic asthma (r = 0.8785, *P* < 0.001). In contrast, no strong correlations were identified between circulating IL-13^+^ILC2s numbers and blood eosinophils counts (r = 0.3247, *P* > 0.05), and FEV1% of predicted (r = − 0.5252, *P* > 0.05). Besides, no significant relationship was found between IL-13^+^ILC2s levels and clinical parameters in HMD-allergic asthmatics (Fig. 5).

### Distinct cytokines expression from IL-25 and/or IL-33-induced Lineage^−^ cells between pollen-allergic and HDM-allergic asthmatics

**Fig. 6:**
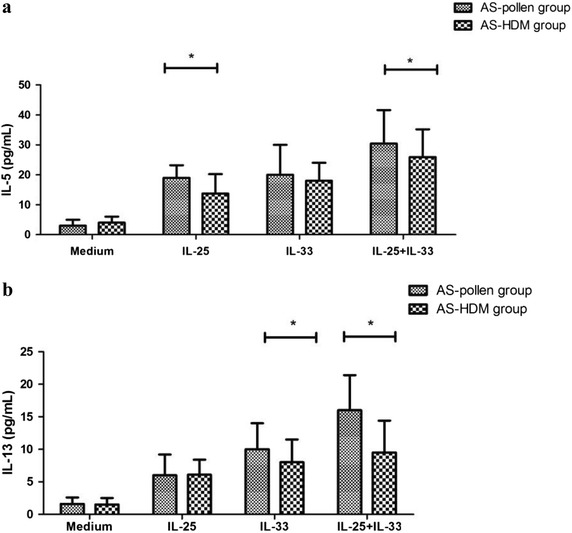
Distinct cytokines expression from IL-25 and/or IL-33-induced Lineage^−^ cells between pollen-allergic and HDM-allergic asthmatics. The Lineage^−^ cell fraction was isolated from subjects with who were mono-sensitized to pollen (AS-pollen group) and with who were mono-sensitized to HDM (AS-HDM group) and then cultured for 7 days with medium, IL-2 (20 U/mL), IL-33 (25 ng/mL) alone, IL-33 (25 ng/mL) alone or a combination of IL-25 and IL-33. The levels of **a** IL-5 and **b** IL-13 in cell-free supernatants were measured by a Milliplex human cytokine array kit. The lower limit of Multiplex tested for detection was IL-5: 0.6 pg/mL, IL-13: 0.7 pg/mL, respectively). *P < 0.05

We performed subsequent mechanistic studies to investigate the potential role of ILC2s as source of type 2 cytokines in the pathogenesis of allergic conditions, and to verify whether this effect was varied among patients sensitized to different types of allergens. Our data showed the Lineage-cells from both mugwort-allergic asthma group and HDM-allergic asthma group could produce significant amounts of IL-5 and IL-13 after the stimulation with IL-33 when compared to IL-2 alone (all *P* < 0.05, respectively), or medium alone (all *P* < 0.05, respectively). Similarly, higher levels of IL-5 and IL-13 were observed in IL-25-stimulated Lineage-cells from both patients subgroups (all *P* < 0.05, respectively). Furthermore, subgroup analysis showed that IL-33 stimulation had a stronger effect on the Lineage-cells from mugwort-allergic asthmatic to release IL-13, whereas a significant elevation in IL-5 release was observed in IL-25-stimulated Lineage-cells from mugwort-allergic asthmatic compared with that of HDM-allergic asthmatics (Fig. 6).

## Discussion

In present study, our date demonstrated that within mugwort pollen season a significantly higher percentage of circulating ILC2s was detected in asthma patient subgroups than healthy controls. Our results are consistent with the previous finding presented by Zhang et al. showing that an increased circulating ILC2s was observed in HDM-allergic patients, indicating the important role of ILC2s in airway allergic reactions. Moreover, a subgroup analysis of asthma patients in present study demonstrated that within pollen season the frequencies of ILC2s were significantly increased in mugwort-allergic asthmatics than that in HDM-allergic asthmatics. When outside the pollen season, an obvious decline trend of circulating ILC2s was observed in pollen-allergic patients, however, no seasonal changes in the proportions of circulating ILC2s were observed HDM-allergic asthmatics. Similarly, Fan et al. had reported that a distinct phenotypic and functional profiles in ILC2s frequencies existed between HDM-sensitized and mugwort-sensitized allergic rhinitis patients, and the main cause for this discrepancy was assumed to be related to the difference in allergenicity between HDM and mugwort [11]. Pollen of mugwort (*Artemisia vulgaris*) is one of the main causes of allergic reactions in late summer and autumn [12]. Among patients suffering from pollinosis, the incidence of allergic disease caused by mugwort pollen is 10–14% [13]. In Northern areas of China, mugwort pollination commonly occurs at the end of July, with the peak pollen period ranging from August to September [14]. Previous studies reported that the incidence of pollinosis was consistent to airborne pollen peak time, in contrast, a negative relationship was observed between airway responsiveness and airborne pollen concentration [15, 16]. Recently, the major allergen in mugwort pollen has been determined, Art v 1, which is a highly glycosylated protein with an apparent molecule mass of 24–28 kDa react with IgE from > 95% of patients allergic to mugwort. In striking contrast to other allergens that contain multiple T cell epitopes, the T cell responses to Art v 1 is characterized by one strong immunodominant epitope [17]. Jahn-Schmid et al. had further characterized the T responses of mugwort allergic patients to natural Artv1 (nArt v 1), demonstrating that all Art v 1-specific T cell clones expressed the CD4+ CD8-TCR α β + phenotype, and the majority exhibited a Th2 cytokine profiles [18]. In contrast, house dust mites (HDM) are a type of common, persistent, and perennial allergen that is present almost everywhere in the world. HMD and their fecal pellets contain several trypsin/chymotrypsin-like enzymes which could directly lead to tissue damage and increase the passage of allergens across the epithelial barrier, these actions might further stimulate allergic reactions through difference pathway, such as greater epithelial cell-derived IL-33 release [19, 20]. IL-33 has been shown to be a potent stimulus for ILC2s activation and migration in vitro, and promote the expansion of ILC2s into the airway in the initiation of HDM-induced Th2 immunity [21]. Although in present study the detail mechanism of increased peripheral blood ILC2s remains unclear, it is possible to estimate that mite allergen exposure and its direct interaction with airway epithelial cells could exert an important effect on the generation of peripheral ILC2s.

We further identified that the sorted ILC2s produced IL-5 and IL-13, and demonstrating there was a positive correlation between IL-13^+^ILC2s numbers and FeNO levels in pollen-allergic asthmatics, however, no similar results were found in HDM-allergic asthma patients. Yi et al. had demonstrated that the tendency of IL-13^+^ILC2s percentages was incrementally higher following the enhancement of asthma control levels, and a strong positive relationship was found between IL-13^+^ILC2s and GINA scores, FeNO level, respectively [22]. A recent study showed that after repeated allergen exposure, a positive feedback between ILC2s and Th2 cell was indispensable for persistent asthma, in which IL-13 produced by ILC2s and Th2 cells further induce IL-33 production and then induce more ILC2 [23]. Therefore, the incidence of IL-13+ ILC2s may provide us with a surrogate marker of the inflammatory status of the disease, and the physiological function of IL13+ ILC2 among asthma patients with distinct clinical characteristic should be further investigated.

Next, we performed subsequent mechanistic studies to investigate whether there were differences in type 2 cytokines production from Lineage-cells among asthmatics sensitized to different allergens, showing that IL-33 can induce a significantly greater release of IL-13 by the Lineage-cells from mugwort pollen-allergic asthmatic compared to that of HDM-allergic asthmatics, whereas a significant elevation in IL-5 release was observed in IL-25-stimualted Lineage^−^cells from mugwort pollen-allergic asthmatic than that from HDM-allergic asthmatics. Although IL-25 and IL-33 were confirmed to be potent type2-inducing cytokines, experimental mouse studies suggest that IL-33 plays a critical role in the rapid induction of airway contraction by stimulating the prompt expansion of IL-13-producing type 2 innate lymphoid cells, whereas IL-25-induced responses are slower and less potent [24]. In current study, although we did not perform an exact comparison in stimulations magnitude between mugwort pollen and HDM, our findings might raise the possibility that distinct cytokine profiles in airway microenvironment among asthma patients with different sensitized patters could exert different effects on the peripheral ILC2s generation.

In summary, we found that during pollen season there was an elevation in ILC2s in peripheral blood, and a positive relationship between ILC2s and FeNO levels among asthmatics with mugwort pollen-allergy. However, a limitation of the study is its relatively small sample size, which precludes us from performing subgroup analysis. Besides, it has not fully explored the upstream signals in driving circulating ILC2s cell generation and maturation. Clearly, clinical studies with different study designs and larger sample size are necessary to answer the above questions, which could provide insights to understand the immunopathology of asthma and to design of new therapeutic strategies.

## Abbreviations

FeNO: fractional exhaled NO
ILC2s: group 2 innate lymphoid cells
EOS: eosinophils
